# On the Physicochemical Characteristics and Applications of an “Undesirable” Pyrenean Thorny Cushion Dwarf: *Echinospartum horridum* (Vahl) Roth

**DOI:** 10.3390/plants9091180

**Published:** 2020-09-11

**Authors:** Pablo Martín-Ramos, Jesús Martín-Gil, Daniel Gómez-García, José Antonio Cuchí-Oterino

**Affiliations:** 1 Instituto Universitario de Investigación en Ciencias Ambientales de Aragón (IUCA), EPS, Universidad de Zaragoza, Carretera de Cuarte, s/n, 22071 Huesca, Spain; 2 Agriculture and Forestry Engineering Department, ETSIIAA, Universidad de Valladolid, Avenida de Madrid 44, 34004 Palencia, Spain; mgil@iaf.uva.es; 3 Instituto Pirenaico de Ecología (CSIC). Avda. Ntra. Sra. de la Victoria 16, 22700 Jaca, Spain; dgomez@ipe.csic.es; 4 Instituto Universitario de Investigación en Ingeniería de Aragón (I3A), EPS, Universidad de Zaragoza, Carretera de Cuarte, s/n, 22071 Huesca, Spain; cuchi@unizar.es

**Keywords:** erizón, ESI-TOF, genkwanin, shrublands, thermal analysis, Pyrenees

## Abstract

Small evergreen shrubs of the family Fabaceae represent a large proportion of current Mediterranean mountain vegetation. Their low pastoral value and tendency for encroachment makes these plants undesirable. In this paper, the thermal and chemical characteristics of *Echinospartum horridum*, a thorny cushion-shaped dwarf shrub native to the French Central Massif and the Pyrenees (particularly dominant in the shrublands of the Pyrenees), have been analyzed with a view to its valorization. Although the higher and lower heating values of the biomass from *E. horridum* met the ISO 17225-2:2014 requirements for its use in pellets, the ash content was slightly above the upper limit, so it would not comply with the normative for its acceptable use as a fuel. Nevertheless, the presence of high added-value flavonoids and lignans in its extracts, which are receiving increasing recent interest as efficient anti-tumor drugs and antivirals, may open the door to the valorization of this shrub for pharmacological applications.

## 1. Introduction

**Figure 1:**
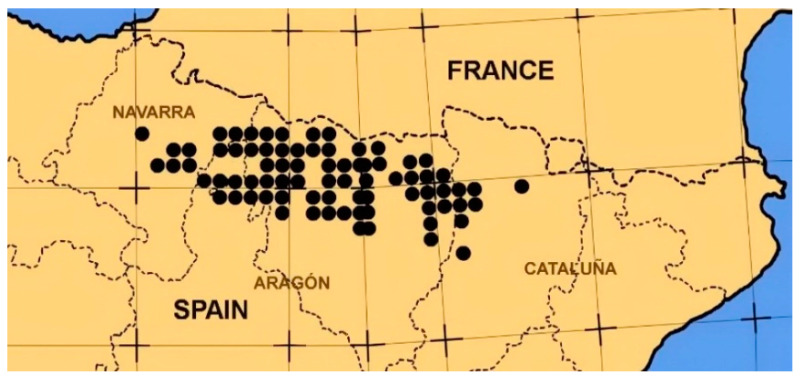
*Echinospartum horridum* distribution in the Pyrenees mountain range.

*Echinospartum horridum*, commonly called “erizón”, “abrizón”, “escarpín” or “tollaga” is a long-lived (up to 30–40 years) perennial shrub of the Fabaceae family, endemic of the south-west extreme of Europe. Its distribution area is restricted to the central-western sector of the Pre-Pyrenees and Pyrenees ranges (from Leoz and Roncal Valleys in Navarra to the Ribagorza in Aragon) and barely crosses the borderline to the French northern slope (Figure 1). It may also be found in a tiny village in the Massif Central in France.

**Figure 2:**
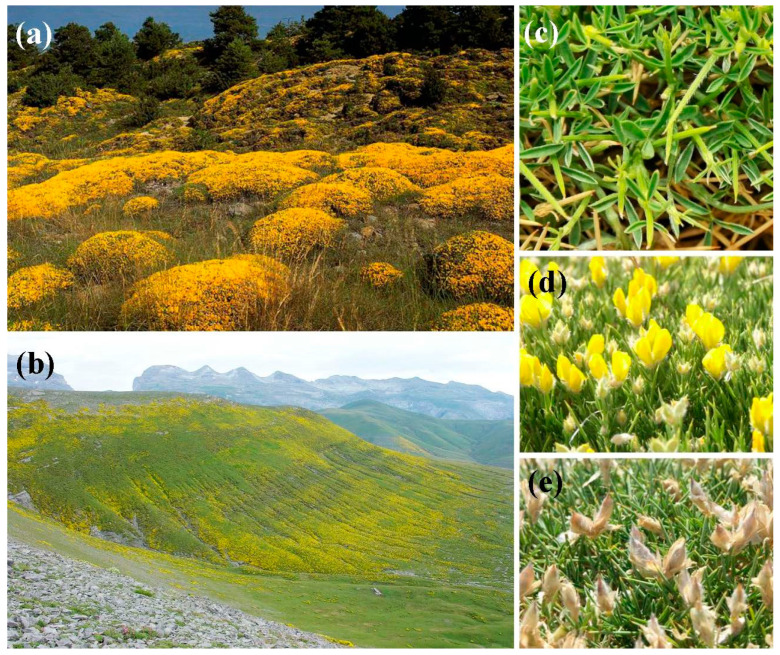
(**a**,**b**) *E. horridum* shrublands in Sierra de las Cutas, Ordesa-Monte Perdido National Park, Huesca, Spain; (**c**) leaves; (**d**) flowers; and (**e**) fruits.

As for its morphology, *E. horridum* is a taprooted, thorny, cushion-shaped shrub with a very dense structure, which can exceed 50 cm in height and 1 m in diameter. Branch and stipule apexes are bristly. The leaf is composed, with three lanceolate, hairy below, early-falling leaflets of only 5–8 × 1 mm. The bright yellow flowers are 1 cm long, solitary or, most often, grouped in pairs. The fruit is a legume covered with silky hairs, 1–2 cm long with 1–3 ovoid and blackish seeds (Figure 2). The flowering period is between June and July, according to altitude.

*E. horridum* is found at between 400 and 2344 m, although its greatest abundance is observed between 1000 and 1700 m [1]. It grows on calcareous or marlstone substrates. Its primary habitat is ridges and rocky slopes, where it forms dense shrublands that constitute permanent communities, i.e., unable to evolve into forest in the short term (Figure 2). Thanks to its deep roots, morphology and phenology, this shrub withstands the summer drought of the environments it occupies, focusing its organogenesis in spring (April and May) [2,3]. The plant is unable to re-sprout, but its seeds germinate vigorously, especially after fire or thinning of vegetation.

In the last few years, an increase of Pyrenean grassland encroachment, mainly due to this shrub, has been reported [4], and prescribed burning and mechanical clearing, in order to control the expansion of the bush, are being carried out. These actions, apart from being costly and sometimes harmful due to the use of machinery in abrupt topographies, are proving ineffective due to the aforementioned germination capacity of this shrub [5]. Studies on burning treatment and the fire’s thermal signature in areas covered by this shrub have been reported by Badía et al. [5,6].

The nutritive value of *E. horridum* is constrained by its high lignin content, which restricts the availability of nutritive palatable pasture barely past the seedling stage [7,8]: only the tender buds and flowers are consumed by sheep and goats. The low pastoral value, together with the abundant spinescence, highlight the difficulty in controlling the expansion of this species via grazing and challenge the suitability of prescribed burning for fighting shrub encroachment [9].

However, although this bush is widely regarded as undesirable, it should be noted that it is able to maintain the soil in environments very prone to erosion and that it fixes atmospheric nitrogen, thus contributing to the improvement of the soils where it is located. Moreover, the leaflets and stems of Genistae (*Echinospartum* spp.) have been reported to be rich in daidzein, formononetin, genistein, 5-*O*-methylgenistein, biochanin A and 5-O-methylbiochanin A isoflavones [10,11].

Since, at present, this shrub is considered a problem and the control of its expansion is ineffective, it seems appropriate to test the possibility of its economic exploitation. The aim of this study has been to investigate its thermal and chemical characteristics with a view to its valorization, in line with other European Union-sponsored studies directed towards the valorization of the underused natural resources of shrublands.

## 2. Results and Discussion

### 2.1. Fuel Characterization and Biomass Combustion Properties

#### 2.1.1. Elemental Analysis

The C, H, and N percentages of *E. horridum* components (wt% of dry material) were in the 48.3–50.3%, 6.51–6.56% and 0.90–2.08% range, respectively (Table 1). These values are in good correspondence with the overall chemical composition reported for other shrubs, such as *C. ladanifer* (47.8% C, 6.4% H and 0.8% N) or *E. arborea* (51.0% C, 6.2% H and 1.0% N) [12].

The distribution of nitrogen content, with maximum values in the leaves and decreasing by half in the stems and branches, is in agreement with that reported by García Rosa [13] for *C. ladanifer* fractions. Regarding the C/N ratio values, those obtained for the leaves (23.2%) are similar to those found for *C. ladanifer* (26.5%), but half of those obtained for *E. arborea* (50.5%) [12].

#### 2.1.2. Higher Heating Values Derived from Elemental Analysis Data

The calculated higher heating values (HHV) for green stems, brown stems and twigs were 19.67, 20.4 and 20.63 kJ·g^−1^, respectively, with a mean value of 20.2 kJ·g^−1^. For comparison purposes, the HHV values for other Mediterranean shrubs, i.e., *C. ladanifer* and *E. arborea*, ranged from 19.2 to 20.5 kJ·g^−1^ and from 19.7 to 21.3 kJ·g^−1^, respectively [14]. Regarding the applicability of the above shrubs as fuels, the European standard for wood pellets (EN 14961-2) establishes that the HHV should be ≥18.82 kJ·g^−1^ [15], so *E. horridum* aerial samples would be compliant.

#### 2.1.3. Component Percentages

Mass fractions of the different constituents and fresh and dry weights for four *E. horridum* samples are reported in Table 2. Weighted average moisture content values ranged from 13.2% to 18.5%, lower than that reported for *C. ladanifer* (26.8%) and *E. arborea* (26%).

The results obtained for dry stems (10.5–15.3%) were close to those reported by Badía et al. [5] (15.6–17.8%), but noticeable differences were detected for green stems (19.6–34.3% vs. 63.3–77.5%) and twigs (14.8–22.8% vs. 32.7–33.7%). Such differences can be justified by the seasonal variability of dry matter content and its relationship with shoot growth and nonstructural carbohydrates [7].

#### 2.1.4. HHV Values Derived from Component Percentages

The overall HHV for *E. horridum* can be estimated taking the mass fraction of each biomass component (Table 3). Given that this shrub has 15–28% of leaves/green stems, average: 21.5% (× 20.53 kJ·g^−1^ = 4.41 kJ·g^−1^); 70–80% of brown stems, average: 75% (× 19.42 kJ·g^−1^ = 14.56 kJ·g^−1^) and 2–5% of twigs, average: 3.5% (× 19.16 kJ·g^−1^ = 6.7 kJ·g^−1^), the weighted mean, i.e., the HHV value, was 25.6 kJ·g^−1^.

#### 2.1.5. Thermal Analysis

**Figure 3:**
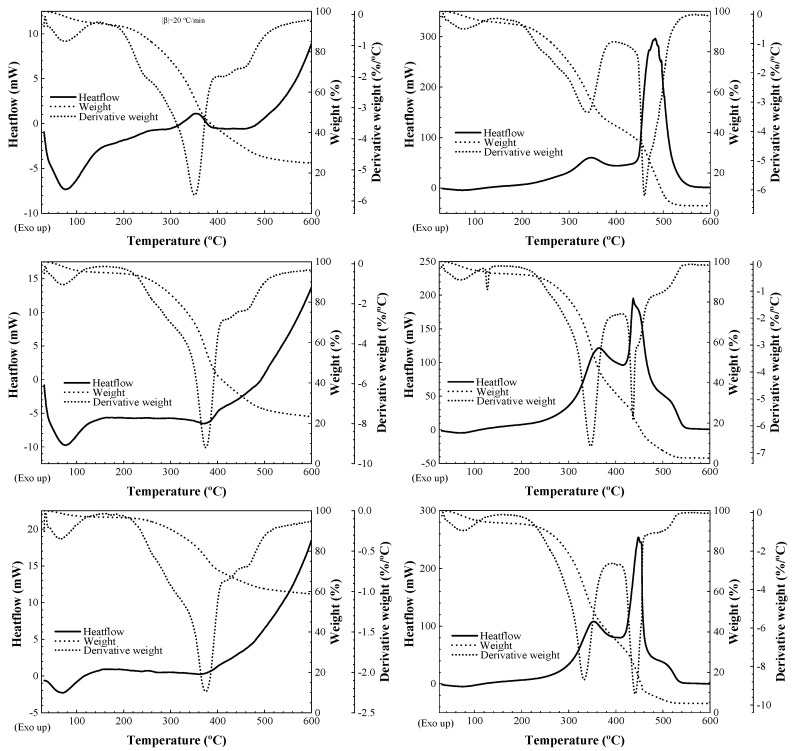
Differential scanning calorimetry (solid line, y-axis on the left), thermogravimetry (dashed line, first y-axis on the right) and derivative thermogravimetry (dotted line, second y-axis on the right) curves of *E. horridum* fractions: (top) green stems or leaves; (center) brown stems; (bottom) twigs. Graphs on the left correspond to thermograms obtained in inert (N_2_) conditions, while those on the right were obtained in oxidative conditions (O_2_). All thermograms were obtained at a heating rate of 20 °C·min^−1^.

The thermogravimetry (TG), derivative thermogravimetry (DTG) and differential scanning calorimetry (DSC) curves for *E. horridum* components are shown in Figure 3, and the temperatures of all thermal effects are summarized in Table 3. The main thermal effects, assigned to the combustion of holocellulose and lignin, are presented in Table 4.

#### 2.1.6. Ash Content

The ash content after heating at 600 °C, in O_2_ atmosphere, was between 2.7% and 3.6% for stems and 4.5% for branches. This would prevent their use as fuels, provided that the threshold ash content value for wood pellets—according to ISO 17225-2:2014 standard [16]—is 2%.

#### 2.1.7. Lower Heating Values

Enthalpy change values obtained from the thermal heating of brown stems and branches (Figure 3) were in the 17.8–18.3 kJ·g^−1^ range. They would correspond to lower heating values (LHV), given that they are in close correspondence with the values expected from the net calorific values of holocellulose and lignin (around 17 and 21 kJ·g^−1^, respectively [17]). The maximum LHV that could be generated by typical biomass is 20.4 kJ·g^−1^ [18] and the relatively high values for *E. horridum* would be in agreement with its high lignin content (215 g·kg^−1^ dry matter) [8]. Thus, LHV values for *E. horridum* would comply with ISO 17225-2:2014 [16]/ENplus [19] requirements (≥16.56 kJ·g^−1^).

### 2.2. Vibrational Spectroscopy Characterization

**Figure 4:**
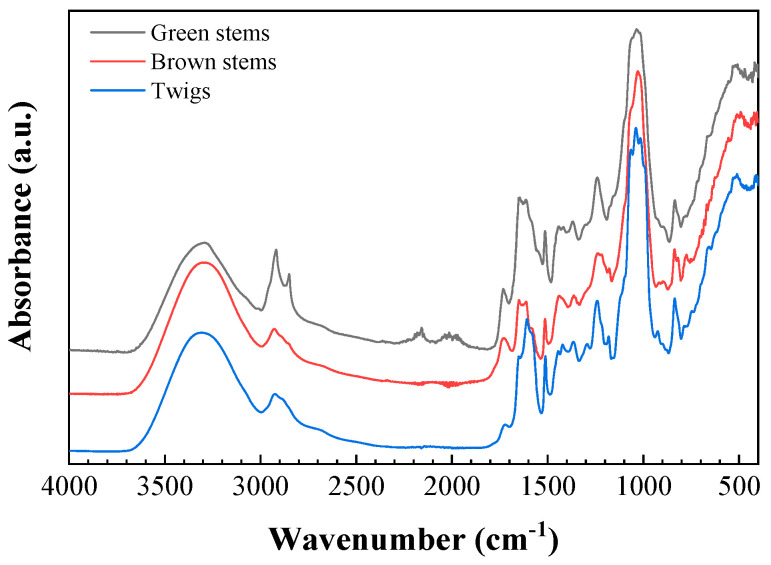
Infrared spectra of the different fractions of *E. horridum*.

Fourier-transform infrared (FTIR) spectra (Figure 4), apart from evidencing the presence of lignin, cellulose and hemicellulose, indicated the presence of the flavone nucleus [20], since they displayed absorption bands at 3289 cm^−1^ (OH), 1640 cm^−1^ (conjugated double bond) and 1613 cm^−1^ (conjugated and chelated γ-pyrone carbonyl), as shown in Table 5.

### 2.3. High Added-Value Products Characterization and Quantification

#### 2.3.1. Identification

**Figure 5:**
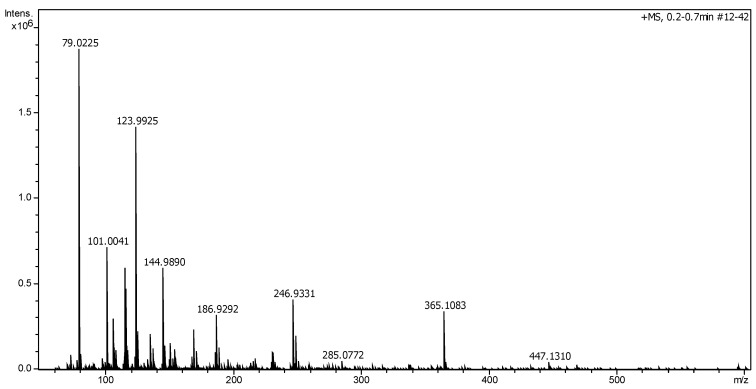
Electrospray ionization quadrupole time-of-flight mass spectrometry (ESI Q-TOF MS) spectrum of *E. horridum* in positive mode.

The electrospray ionization quadrupole time-of-flight mass spectrometry (ESI Q-TOF MS) spectrum in positive ionization mode featured peaks at *m/z* 447, 365, 285, 247, 187, 145 and 124 (Figure 5), which led to the identification of flavonoids and other compounds present in *E. horridum*.

**Figure 6:**
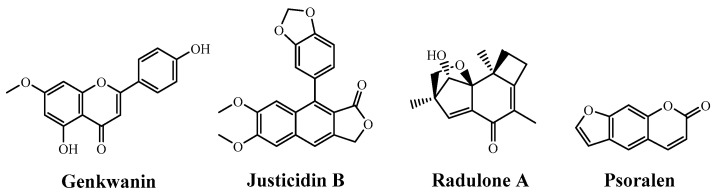
From left to right: genkwanin, justicidin B, radulone A and psoralen.

While in hydrolyzed leaves of *Erinacea anthyllis* Lynk, the identified flavonoids were daidzein, genistein and isoprunetin [10], in *E. horridum,* the main flavonoid would be genkwanin, [M+H]^+^ *m/z* = 285, a monomethoxyflavone that derives from apigenin (Figure 6), which is structurally close to isoprunetin (5-*O*-methylgenistein). Genkwanin shows inhibitory effects on breast cancer resistance protein (BCRP) [25] and inhibition of African swine fever virus infection [26]. Provided that the anhydro-glucopyranoside moiety, Glc*p*, is exactly 162 Da, the precursor peak at *m/z* = 447 should be assigned to its 4′-*O*-*β*-d-glucopyranoside derivative.

Other interesting natural chemical components present in *E. horridum* would be justicidin B (an arylnaphthalene lignin, [M+H]^+^ *m/z* = 365), with applications in cancer chemotherapy [27] and as an antiviral [28]; the illudane type-sesquiterpene radulone A, [M+H]^+^ *m/z* = 247; and the linear furanocoumarin psoralen, [M+H]^+^ *m/z* = 186, with activity against breast cancer [29] (Figure 6).

The peak at *m/z* = 124 can be associated to the *m/z* = 285 fragment by neutral loss of a monosaccharide molecule (−161); that at *m/z* = 101 can to correspond to the *m/z* = 145 fragment ion via loss of a CO_2_ group from the carboxylic acid; and the peak *m/z* = 79 to [C_6_H_7_]^+^. Finally, the peak at *m/z* = 145 can be associated either to a sugar fragment [C_7_H_13_O_3_]^+^ or to protonated *α*-naphtol, [C_10_H_9_O]^+^.

#### 2.3.2. Genkwanin Quantification

For the economic justification of the pharmacological use of the studied shrub plant, if the isolation of the aforementioned compounds is considered, information on their content is very important from the point of view of the efficiency of the process. Hence, genkwanin content was determined for the leaves/green stems fraction by HPLC analysis. The yield attained for this bioactive compound using methanol extraction under heating and stirring was 0.21 ± 0.04 mg/g (*n* = 3). This content is 25% lower than the one reported by Qi et al. [30] for *Equisetum palustre* L. (0.28 mg/g) and an order of magnitude lower than the values found by Lin et al. [31] in *Daphne genkwa* Sieb et Zucc. flowers (3.7 mg/g).

### 2.4. Opportunities for Valorization

The shrub species under study, *E. horridum*, yielded LHV and HHV values compliant with the requirements of solid biofuel standards. Nonetheless, its biomass ash content was slightly above the maximum established in the European normative for wood pellets (2%), which would preclude their utilization as fuelwood in rural district heating. Since the ash content depends on the dryness of the plants to be used as fuel, only old specimens (entirely brown stems) should be harvested in order to meet the regulatory requirements.

An alternative application, with a higher added-value, would be the one derived from the pharmacological use of some flavonoids (genkwanin) and lignans (justicidin B) present in its composition. These components, upon administration, show dominant anti-tumor and antiviral activities. At present, genkwanin market price exceeds 200 €/10 mg, which could make the felling and harvesting of these shrubs a profitable activity if higher extraction yields can be attained. It should be taken into consideration that the amount reported above (0.21 mg/g) may be regarded as a lower bound estimate, given that genkwanin-5-*O*-β-d-glucopyranoside was not quantified and that the extraction conditions had not been optimized.

The use of modern extractive approaches, such as dynamic maceration process [32] or ionic liquid analogs (deep eutectic solvents) as extractive solvents [33], negative pressure cavitation-assisted extraction with macroporous resin enrichment [30], microwave-assisted extraction, high-speed countercurrent chromatography [34], etc. should be explored to find an effective preparative method for the isolation and purification of flavonoids from *E. horridum*.

## 3. Material and Methods

### 3.1. Sampling

The *E. horridum* samples under study were collected in Monrepós pass, Huesca, Spain (42°18.9890′ N, 0°16.6929′ W, 1191.2 m.a.s.l.) in May 2019. They corresponded to healthy individuals and their characteristics were akin to those of the rest of the population. Just the aerial part was used in the study, given that excavation of the root system would not be viable for the commercial exploitation of this shrub.

### 3.2. Fuel Characterization and Biomass Combustion Properties

The selected samples were processed at the Escuela Politécnica Superior facilities (Universidad de Zaragoza, Huesca, Spain), separating the various fractions and weighing them (fresh weight). Subsamples from each fraction, dried in an oven at 102 ± 2 °C until constant weight was attained (at which the water content was assumed to be zero), were used for the determination of the dry matter content.

Prior to the physicochemical characterization, each dried biomass fraction was ground into powder in a ball mill, homogenized, and sieved (1 mm mesh).

Elemental (CHNS) analyses of the dry ground samples were performed using a TruSpec Micro (LECO, St. Joseph, MI, USA) apparatus.

Thermal analysis was conducted with a TG-DSC2 instrument (Mettler Toledo; Columbus, OH, USA). The samples were heated at a rate of 20 °C·min^−1^ from 30 to 600 °C, under both N_2_ and air flow (20 cm^3^·min^−1^). The ash content was estimated from the residue obtained after heating at 600 °C, in agreement with the usual pyrolysis temperature conditions in oxygen bomb calorimeters [35].

#### Calorific Values Calculation

The calculation of calorific values from elemental analysis data was carried out according Equation (1) [36]:

HHV = (0.341 × %C) + (1.322×%H) − 0.12(%O + %N),(1)

where HHV is the heating value for the dry material, expressed in MJ/kg, and %C, %H, %O and %N are the mass fractions, expressed in wt% of dry material. This formula gives acceptable results for biomass, according to the UK’s Combined Heat and Power Quality Assurance program [37].

### 3.3. Vibrational Characterization

The infrared vibrational spectra were acquired using a Nicolet iS50 FTIR spectrometer (Thermo Scientific; Waltham, MA, USA), equipped with an in-built diamond attenuated total reflection (ATR) system. The spectra were collected with a 1 cm^−1^ spectral resolution over the 400–4000 cm^−1^ range, taking the interferograms that resulted from co-adding 64 scans.

### 3.4. Extraction Process and High-Added Value Products Characterization and Quantification

#### 3.4.1. Extraction Process

The extraction process was analogous to that used by Lin et al. [31]. A sample of *E. horridum* leaves/green stems fraction (1.0 g) was extracted three times with 70% methanol at 80 °C for one hour. The extracts were combined and methanol was added to 150 mL. This solution was filtered through a 0.45 μm filter before use.

#### 3.4.2. Electrospray Ionization Quadrupole Time-of-Flight Mass Spectrometry (ESI-Q-TOF MS) Characterization

For the identification of the various compounds present in the extract, the high-resolution mass spectrum was measured on a Maxis Impact spectrometer (Bruker Daltonik GmbH, Bremen, Germany) using direct infusion ESI Q-TOF MS. The spectrometer was operated in linear positive mode in the *m/z* range of 50–600 Da, under the following conditions: 3.5 kV potential between spray needle and orifice, 0.4 bar nebulizer pressure, 3.0 L·min^−1^ drying gas flow rate at 200 °C, and 3.0 µL·min^−1^ sample flow rate [38]. The analysis was outsourced at the Laboratorio de Técnicas Instrumentales facilities of Universidad de Valladolid.

#### 3.4.3. Genkwanin Quantification

In order to estimate the content of the most important high-added value product (genkwanin) detected in *E. horridum* extract, high-performance liquid chromatography (HPLC) analysis was conducted using an Agilent 1200 HPLC system (Agilent Technologies; San Jose, CA, USA). The chromatographic separation was carried out on a Curosil^TM^-PFP (pentafluorophenyl) column (250 × 4.6 mm i.d., 5 μm, Phenomenex, Torrance, CA, USA), whose temperature was kept at 30 °C. The gradient elution program was the one described by Qi et al. [30], using 0.1% formic acid aqueous solution and methanol. An injection volume of 5 μL and a flow rate of 1.0 mL·min^−1^ were used. The detection wavelength was 330 nm. Genkwanin (≥98%) supplied by Sigma-Aldrich was taken as a reference standard.

## 4. Conclusions

Based on the thermal characterization results, *Echinospartum horridum* biomass does not comply with ISO 17225-2:2014 requirements for its valorization as a fuel. Even though its HHV and LHV calorific values (25.6 and 18.3 kJ·g^−1^, respectively) meet the requirements for its use in pellets, the ash content (2.7–4.5%, depending on the specific fraction) exceeds the established limit (<2%), so it would not comply with the normative for its acceptable use as a fuel. Nonetheless, the presence of genkwanin, justicidin B, radulone A and psoralen high added-value by-products in its extracts may offer a promising alternative for the valorization of this neglected and underutilized natural resource of Pyrenean shrublands in pharmacology.

## Figures and Tables

**Table 1:** Elemental (CHNS) composition (wt%) of *E. horridum* fractions.

Sample	%C	%H	%N	%S	C/N Ratio
Leaflets/green stems	48.27	6.53	2.08	0.00	23.2
Brown stems	49.88	6.51	0.90	0.00	55.4
Little branches (twigs)	50.27	6.56	1.05	0.00	47.9
Sulfamethazine standard	51.86	5.05	20.13	11.41	
Sulfamethazine sample	51.75	5.09	20.49	11.83	

**Table 2:** Moisture values for *E. horridum.*

Sample	Fraction	Mass Fraction (wt%)	Fresh Weight (g)	Dry Weight (g)	Moisture (g)	Moisture Content (wt%)	Moisture Content (wt%, Weighted Average)
1	Green stems	15	73.94	55.07	18.87	34.27	18.50
	Brown stems	80	119.1	103.33	15.78	15.27	
	Twigs	5	51.66	42.07	9.59	22.80	
2	Green stems	21	63.17	48.73	14.44	29.63	16.40
	Brown stems	75	97.68	86.73	10.95	12.63	
	Twigs	4	37.41	31.78	5.63	17.72	
3	Green stems	28	30.21	25.12	5.09	20.26	13.50
	Brown stems	70	69.50	62.75	6.75	10.76	
	Twigs	2	15.51	13.51	2.00	14.80	
4	Green stems	27	30.80	25.75	5.05	19.61	13.22
	Brown stems	70	64.21	58.09	6.12	10.54	
	Twigs	3	20.38	17.24	3.14	18.21	

**Table 3:** Weight loss (%) and thermal effects temperatures for *E. horridum* constituents.

Fraction	Purge Gas	Weight Loss (%)	DTG (°C)					DSC (°C)				
Leaves/ green stems	N_2_	24.9	75.4	350.4	420	458		75.4 endo	354 exo			
	O_2_	3.65	78.5	339.1		460		73.5	347 exo		483 exo	
Brown stems	N_2_	23.3	70	374.7	425.5	463.3		76.6 endo		405 exo		
	O_2_	2.7	70	347.3	437	450.5	512	68	365 exo		465 exo	515.5 exo
Little branches (twigs)	N_2_	58.9	66	374.5	422	462.7		69 endo				
	O_2_	4.5	73	333.0	441		505		352 exo		448 exo	505 exo

endo: endothermic effect, exo: exothermic effect.

**Table 4:** Main exothermic effects in the DSC curves of *E. horridum* fractions associated with holocellulose and lignin combustion. Those of *C. ladanifer* and *E. arborea* are included for comparison purposes [14]. T_peak_ stands for exotherm peak temperature.

Species, Fraction, Purge Gas	Holocellulose (Cellulose + Hemicellulose) T_peak_ (°C)	Lignin T_peak_ (°C)
*E. horridum*, green stems, O_2_	347	483
*E. horridum*, brown stems, O_2_	365	465
*E. horridum*, twigs, O_2_	352	448
*C. ladanifer*, air	365	455
*E. arborea*, air	376	527

**Table 5:** Main absorption bands in the infrared spectra shown in Figure 4, together with their assignments [21,22,23,24].

Wavenumber (cm^−1^)	Vegetal Component	Bonds	Assignments
3288–3308		OH	
2917–2927	Cellulose, hemicellulose, lignin	CH stretch	
2849–2850	Cellulose, hemicellulose, lignin	CH stretch	Symmetric CH_2_ valence vibration
1727–1733	Hemicellulose, lignin	C=O	Ester linkage of the carboxylic group of p-coumaric and ferulic acids
1648–1652	Flavone nucleus		Conjugated double bond
1621–1625	Cellulose, lignin	O−H, C−O	O–H and conjugated C–O
1612–1614	Flavone nucleus	C=O	Conjugated and chelated γ-pyrone carbonyl
1541–1558			Aromatic ring
1514–1516	Lignin	C=C	Stretching vibrations of aromatic structure
1437–1443	Saccharide backbone	C−H	Alkane deformation relating to CH and CH_2_, consistent with the saccharide backbone
1417		C=C	C=C ring stretching
1363–1370	Cellulose	C−H	In-plane bending vibration of the C−H and C−O groups of the hexose ring
1316	Cellulose, hemicellulose, lignin	C-O, CH_2_	Condensation of the guaiacyl unit and syringyl unit; syringyl unit and CH_2_ bending stretching; CH_2_ rocking vibration
1237–1240	Lignin	C−H	C−C plus C−O plus C=O stretching (OH plane deformation, also COOH)
1178		C−H	
1151	Cellulose	C−H	C–O–C asymmetric valence vibration, C=O stretching in aliphatic group
1027–1035	Cellulose, hemicellulose, lignin	C−O, C=C and C−C−O	Aromatic C–H in plane deformation; plus C−O deformation in alcohols; plus C=O stretch (unconjugated)
